# Willingness of Nepalese medical and nursing students to volunteer during COVID-19 pandemic: A single-centered cross-sectional study

**DOI:** 10.1016/j.amsu.2021.103056

**Published:** 2021-11-18

**Authors:** Parag Karki, Lee Budhathoki, Manoj Khadka, Swojay Maharjan, Subodh Dhakal, Subashchandra Pokharel, Anita Poudel, Pooja Rokaya, Udit Raut, Sushma Rayamajhi

**Affiliations:** a Department of Internal Medicine, Shree Birendra Hospital, Kathmandu, Nepal; b Nepalese Army Institute of Health Sciences, 44600, Kathmandu, Nepal

**Keywords:** COVID-19, Cross-sectional study, Medical students, Nursing students, Volunteers

## Abstract

**Background:**

Medical students, being more familiar with medical situations, can play a vital role as volunteers during medical crises like mass casualty emergencies and epidemics. This study was conducted to know the willingness of medical and nursing students to volunteer during the coronavirus disease-19 (COVID-19) pandemic.

**Materials and methods:**

This cross-sectional study was conducted among undergraduate medical and nursing students of a medical college in Kathmandu. A proportionate stratified random sampling technique was used. A pretested, self-administered questionnaire was emailed to participants and the data were collected from 8th July to July 29, 2021 via the Google forms, extracted to the Google sheets, and then analyzed using Statistical Package for Social Sciences (SPSS) version 16.

**Results:**

Out of 288 randomly selected participants, a total of 261 valid responses were obtained, giving a response rate of 90.6%. The majority (n = 203, 77.8%) of the participants were willing to volunteer. The most preferred area of work during volunteering was clinical care of the COVID-19 patients (n = 74, 36.5%), followed by involvement in health education and awareness-raising activities (n = 63, 31%). Among those not willing to volunteer (n = 58, 22.2%), the most commonly reported reason was the lack of adequate training and skills (n = 23, 40%).

**Conclusion:**

Since the majority of medical and nursing students were willing to volunteer during the times of COVID-19, they can be of great help as a human resource in case of shortage of healthcare professionals. As lack of training and adequate skills was the main reason for those not willing to volunteer, we recommend the provision of adequate training and skills before deploying students as volunteers during health crises like COVID-19.

## Highlights

• This study aims to know the willingness of medical and nursing students to volunteer during the COVID-19 pandemic.
• The majority of students were willing to volunteer, so they can help during the shortage of healthcare professionals.
• The most commonly preferred area of work by the students was clinical care of the COVID-19 patients.
• We recommend that the students should be adequately trained and skilled before being deployed in health crises like COVID-19.

## 1 Introduction

Coronavirus disease-2019 (COVID-19) has placed a significant burden on the global healthcare system, prompting the World Health Organization (WHO) to declare it a pandemic on March 11, 2020 [1]. Various public health responses like travel restrictions, physical distancing, quarantining, social isolation, and contact tracing were used to control the pandemic [2]. Despite various public health response strategies to halt the pandemic, challenges like shortage of resources including financing, personal protective equipment (PPE), and respiratory devices were creating the obstacle, especially in the low-income countries [3,4]. Besides these, another important barrier was the lack of health care personnel [4].

For every 1000 people, WHO recommends a minimum of 2.28 health care personnel (doctors, nurses, and midwives) [5,6]. As per World Health Statistics 2018, for every 1000 people, there were 0.8 physicians and 2.1 nurses and midwives in India while 0.6 physicians and 2.0 nurses and midwives in Nepal [6,7]. So, the South Asian countries including India and Nepal were already short of healthcare personnel before the pandemic began, and this shortfall was more evident with the emergence of the COVID-19 pandemic, increasing the work burden for the healthcare personnel. Thus, the recruitment of healthcare personnel and other human resources is crucial to fight the pandemic [8]. Medical students, being more oriented to medical situations are one of the alternatives for assisting overworked health professionals. During the influenza pandemic in 1918, medical students were involved in patient care to aid the medical personnel [9]. As a result, medical students serving as volunteers might be a vital response to the COVID-19 pandemic.

So, the primary objective of this study was to evaluate the medical and nursing students' willingness to volunteer during the COVID-19 pandemic. The secondary objectives were to determine the students' risk perception and attitude towards volunteering, to assess the students' reasons for volunteering, and to know the areas where medical and nursing students are willing to volunteer during the COVID-19 pandemic.

## 2 Methods

### 2.1 Study design and setting

We conducted a cross-sectional study among the undergraduate medical and nursing students of the Nepalese Army Institute of Health Sciences (NAIHS), Kathmandu, Nepal. We used the proportionate stratified random sampling method. The study participants were selected randomly using the computer random number generator maintaining an equal proportion of students from both medical and nursing groups of students. Equal proportion of students from each year within the groups and an equal proportion of males and females in each year were selected. The questionnaire was emailed to the participants and the responses were collected from 8th July to July 29, 2021 via Google forms.

### 2.2 Population and study sample

Undergraduate medical and nursing students studying in the Nepalese Army Institute of Health Sciences who gave consent to participate were included in the study.

The sample size for the study was calculated using Cochran's formula:

$$\begin{array}{l} \text{Sample}\;\text{size}\;\left(\text{n}\right)\;=\;{\text{Z}}^{2}\;x\;\left(p\;x\;q\right)/e^{2}\\ \;=3.8416\;x\;(0.5\;x\;0.5)/0.0025\\ \;=385 \end{array}$$

where,

z = confidence interval at 95%, z: 1.96
p = prevalence taken as 50%; q = 1-p;
e = margin of error, 5%

Total undergraduate medical and nursing students in NAIHS (N) = 790 (540 from MBBS and 250 from nursing)

Adjustedsamplesize(n')=n/[1+{(n-1)/N}]=385/[1+(384/790)]=259

Upon dividing this minimum sample size between two groups proportionately, we allocated 68.4% sample size for MBBS group (i.e. 177 students) and 31.6% sample size for nursing group (i.e. 82 students).

Considering 10% as a non-response rate, the required sample size became 288 (197 for the MBBS group and 91 for the nursing group). The questionnaire was emailed to 288 participants (197 from MBBS and 91 from Nursing), among which only 261 responded giving a response rate of 90.62%.

### 2.3 Study instrument

We used a self-administered questionnaire containing 30 items. It contained 8 items for demographic details and history, 5 items for risk perception, 12 items for attitude towards volunteering, and 5 items for willingness test. The questionnaire was developed after extensive literature review in the English language and was pre-tested among 5% of the study sample, modified accordingly, and then emailed to the study participants.

### 2.4 Statistical analysis

The data collected via the Google forms were extracted to Google sheets, cleaned in Microsoft Excel, and then imported and analyzed using SPSS (Statistical Package for Social Sciences) version 16. We used the Kolmogorov-Smirnov (K–S) test and the Shapiro-Wilk test to assess the normality of the data distribution. The data distribution was normal if the significant value of the test is greater than 0.05 and non-normal if the value is below 0.05. Our data were non-normal so we used non-parametric tests using the median and Inter-Quartile Range (IQR) as a measure of central tendency and dispersion respectively. Spearman's rho was used to check the correlation between all possible non-categorical variables. A Pearson's Chi-square test was used to check the association between different categorical variables and willingness to volunteer. The variables that had an association with willingness to volunteer (at p < 0.05) were included in binomial logistic regression analysis. To find an association between different categorical variables and continuous variables like risk perception and attitude towards volunteering, Mann Whitney *U* test, and Kruskal Wallis One Way ANOVA were used, depending upon the number of categories in the categorical variables.

### 2.5 Ethical consideration

The Institutional Review Committee of NAIHS approved the study with reference number 432. All the participants were informed about the study and its objectives during the time of data collection. A consent form was incorporated into the questionnaire itself. So, all the participants filling the form are understood to have provided consent to participate in the study.

The manuscript has been reported in line with the STROCSS criteria [10]. It has also been registered with Research Registry at http://www.researchregistry.com/on November 6, 2021 and the unique identifying number is researchregistry7338.

## 3 Results

### 3.1 Socio-demographic characteristics

A total of 261 valid responses were obtained from 288 randomly selected participants; with a response rate of 90.6%. The mean (SD) age of the respondents was 22.31 (2.01) years, ranging from 18 to 30 years. Majority of the participants were females (n = 152, 58.2%). Out of the valid respondents, 179 (68.6%) were pursuing education in medicine (MBBS) and 82 (31.4%) were in nursing. Among the 179 participants studying medicine, 109 (60.9%) were in the clinical years (third, fourth, and fifth years). Only 68 (26.1%) of 261 respondents were studying on scholarship and among them, 25 (37%) had a scholarship from the Ministry of Education (MoE); the rest of them being funded fully or partially by the Nepalese Army or other institutions.

Interestingly, 92 (35.2%) participants reported having experience of volunteering in healthcare services and 83 (31.8%) participants had themselves or family members infected with COVID-19. However, a substantial number of respondents (n = 219, 83.9%) were fully vaccinated against COVID-19 by the time of data collection (Table 1).

**Table 1:** Socio-demographic characteristics of the respondents (N = 261).

Variables	N (%)	Willingness to volunteer, N (%)		P- value
		Likely; 203 (77.8)	Unlikely; 58 (22.2)	
1. Gender				
Male	109 (41.8)	78 (71.6)	31 (28.4)	0.041
Female	152 (58.2)	125 (82.2)	27 (17.8)	
**2. Female respondents**				
From MBBS	70 (46.1)	60 (85.7)	10 (14.3)	0.300
From Nursing	82 (53.9)	65 (79.3)	17 (20.7)	
**3. Age group**				
Less than 20	13 (5.0)	9 (69.2)	4 (30.8)	
20–23	133 (51.0)	104 (78.2)	29 (21.8)	0.820
23–26	102 (39.1)	79 (77.5)	23 (22.5)	
More than 26	13 (5.0)	11 (84.6)	2 (15.4)	
**4. Educational program**				
MBBS	179 (68.6)	138 (77.1)	41 (22.9)	0.695
Nursing	82 (31.4)	65 (79.3)	17 (20.7)	
**5. Year of study**				
First year	43 (16.5)	24 (55.8)	19 (44.2)	
Second year	45 (17.2)	35 (77.8)	10 (22.2)	
Third year	76 (29.1)	64 (84.2)	12 (15.8)	0.003
Fourth year	61 (23.4)	48 (78.7)	13 (21.3)	
Fifth year	36 (13.8)	32 (88.9)	4 (11.1)	
**6. Level of MBBS students**				
Preclinical year	70 (39.1)	49 (70.0)	21 (30.0)	0.070
Clinical year	109 (60.9)	89 (81.7)	20 (18.3)	
**7. Status of MoE scholarship**				
No MoE scholarship	236 (90.4)	183 (77.5)	53 (22.5)	0.779
MoE scholarship	25 (9.6)	20 (80.0)	5 (20.0)	
**8. Prior volunteering experience**				
Yes	92 (35.2)	72 (78.3)	20 (21.7)	0.890
No	169 (64.8)	131 (77.5)	38 (22.5)	
**9. Past COVID-19 infection to them or their family member**				
Yes	83 (31.8)	66 (79.5)	17 (20.5)	0.644
No	178 (68.2)	137 (77.0)	41 (23.0)	
**10. Full vaccination against COVID-19**				
Yes	219 (83.9)	172 (78.5)	47 (21.5)	0.499
No	42 (16.1)	31 (73.8)	11 (26.2)	

### 3.2 Risk perception and attitude towards volunteering

Responses were quantified using a five-point Likert scale, with a total possible score ranging from 5 to 25 for items assessing risk perception and 12 to 60 for items assessing attitude towards volunteering. But the actual total scores of respondents' risk perception ranged from 11 to 25, with a median (IQR) value of 20 (18–21) and 21 to 56 for attitude, with a median (IQR) value of 41 (38–44). Responses from 5 items of risk perception and 12 items of attitude, each was grouped into 3 categories: low-risk perception, indifferent and high-risk perception; and negative attitude, indifferent and positive attitude, respectively. Out of 261 respondents, 237 (90.8%) perceived the COVID-19 pandemic as a high-risk condition and 212 (81.2%) respondents had a positive attitude towards volunteering. However, 13 (5%) participants perceived it as a low-risk situation and 36 (13.8%) participants had a negative attitude towards volunteering during the COVID crisis (Table 2).

**Table 2:** Risk perception and attitude findings of the participants.

Statements	Strongly Disagree n (%)	Disagree n (%)	Neutral n (%)	Agree n (%)	Strongly Agree n (%)
Risk Perception					
1. I think the disease is dangerous and highly infectious	–	7 (2.7)	29 (11.1)	97 (37.2)	128 (49.0)
2. I am at risk of being infected as a volunteer	5 (1.9)	21 (8.0)	57 (21.8)	104 (39.8)	74 (28.4)
3. I do not think strict lockdown is necessary for such pandemic	69 (26.4)	84 (32.2)	56 (21.5)	38 (14.6)	14 (5.4)
4. I think the disease has got no definitive cure till date	13 (5.0)	25 (9.6)	60 (23.0)	77 (29.5)	86 (33.0)
5. I am confident I will not catch this disease by any chance even if I volunteer	94 (36)	82 (31.4)	59 (22.6)	22 (8.4)	4 (1.5)
**Attitude towards volunteering:**					
1. I have a moral sense of duty to volunteer as a person	5 (1.9)	3 (1.1)	38 (14.6)	111 (42.5)	104 (39.8)
2. I am not capable of volunteering in any pandemic	84 (32.2)	109 (41.8)	51 (19.5)	11 (4.2)	6 (2.3)
3. Volunteering during pandemic is a form of learning experience for students	2 (0.8)	12 (4.6)	42 (16.1)	101 (38.7)	104 (39.8)
4. Government cannot invite medical and nursing students as volunteers	46 (17.6)	98 (37.5)	80 (30.7)	21 (8.0)	16 (6.1)
5. Helping the people of my community brings a great pleasure and joy to me	3 (1.1)	4 (1.5)	15 (5.7)	75 (28.7)	164 (62.8)
6. I do not think I will have priority access to scarce resources if I get infected	9 (3.4)	23 (8.8)	97 (37.2)	83 (31.8)	49 (18.8)
7. I will not mind staying away from my near ones for some time if I volunteer	5 (1.9)	14 (5.4)	43 (16.5)	110 (42.1)	89 (34.1)
8. People will not admire and recognize my work as a volunteer	33 (12.6)	87 (33.3)	80 (30.7)	39 (14.9)	22 (8.4)
9. I am confident that I can convince my parents for allowing me to volunteer during the pandemic	12 (4.6)	38 (14.6)	64 (24.5)	90 (34.5)	57 (21.8)
10. It would be better if government properly mobilizes private clinicians instead of hiring students as volunteers	8 (3.1)	25 (9.6)	90 (34.5)	80 (30.7)	58 (22.2)
11. I think students should volunteer only if monetary incentives are given to them	21 (8.0)	37 (14.2)	83 (31.8)	65 (24.9)	55 (21.1)
12. It would be good to volunteer only in severe shortage of healthcare workers	11 (4.2)	38 (14.6)	57 (21.8)	90 (34.5)	65 (24.9)

We also checked for an association of different variables with risk perception and attitude of respondents towards volunteering. It was found that the distribution of the total score of risk perception was different for different levels of medical studies (p = 0.015) and the total score of attitude was different across different years of study (p = 0.020). The obvious association between the willingness of respondents to volunteer and risk perception (p = 0.024) and attitude towards volunteering (p < 0.001) was also demonstrated (Table 3).

**Table 3:** Association of different variables with risk perception and attitude towards volunteering.

Variables	P- value for risk perception	P- value for attitude towards volunteering
1. Gender^a^	0.646	0.482
2. Female respondents from different programs^a^	0.484	0.109
3. Age group^b^	0.248	0.232
4. Educational program^a^	0.754	0.092
5. Year of study^b^	0.252	**0.020**
6. Level of study^a^	**0.015**	0.133
7. Status of MoE scholarship^a^	0.787	0.606
8. Previous volunteering experience^a^	0.605	0.091
9. History of past COVID-19 infection^a^	0.492	0.110
10. Vaccination status^a^	0.139	0.303
11. Willingness to volunteer^a^	**0.024**	**<0.001**

a using Mann Whitney *U* test.

b using Kruskal-Wallis One way ANOVA test.

We found a weak positive correlation between respondent's age and risk perception (r = 0.145, p = 0.019) and a weak negative correlation between risk perception and attitude (r = −0.123, p = 0.048). We also noticed a weak positive correlation between respondents' attitudes and the likelihood score of respondents willing to volunteer (r = 0.359, p < 0.001) (Table 4).

**Table 4:** Correlation between different variables using Spearman correlation coefficient.

Variables	Spearman Correlation Coefficient (r)	P- value
Age – Risk perception	0.145	0.019
Risk Perception – Attitude	−0.123	0.048
Age – Attitude	0.007	0.911
Attitude – Likelihood strength to volunteer	0.359	<0.001

### 3.3 Willingness to volunteer

Majority of the respondents were willing to volunteer (n = 203, 77.8%) during COVID-19 pandemic; 78 (38.4%) were males and 125 (61.6%) were females. Those who were willing to volunteer, when given a likelihood scale of 1–5, the highest number of respondents selected 4 (n = 84, 41.4%) and 33 (12.6%) showed the strongest likelihood of volunteering by selecting 5. Although 169 (64.8%) participants did not have any prior healthcare volunteering experience, 131 (77.5%) of them were willing to volunteer. Out of 25 respondents with government (MoE) scholarship, the majority (n = 20, 80%) were willing to volunteer. We found gender and year of study were two variables associated with willingness to volunteer (Table 1).

A significant difference was observed between males and females regarding willingness to volunteer (⎟^2^ = 4.187, p = 0.041). When binomial logistic regression analysis was performed, it was found that females were 1.84 (95% CI: 1.02–3.31, p = 0.042) times more likely to volunteer than males. Willingness to volunteer was significantly different in different years of study (⎟^2^ = 16.422, p = 0.003). The odds of willing to volunteer were highest in fifth-year students (OR = 6.33, 95% CI: 1.90–21.05, p = 0.03) as compared to other year students (Table 5).

**Table 5:** Logistic regression analysis on factors associated with willingness to volunteer.

Variables	Willingness to	volunteer	P- value
	Odds Ratio (OR)	95% CI	
1. Gender			
Male	Ref.		
Female	1.84	1.02–3.31	0.042
2. Year of study			
First year	Ref.		
Second year	2.77	1.09–6.99	0.031
Third year	4.22	1.78–9.99	0.001
Fourth year	2.92	1.24–6.90	0.014
Fifth year	6.33	1.91–21.05	0.003

Regarding the motivating factors behind volunteering, 97 (47.8%) out of 203 willing candidates believed that it was an opportunity to serve mankind and 69 (34%) were motivated by the thought that they would learn new things from the volunteering experience. When asked about the most preferred area of work during volunteering, the highest number of the willing participants were interested in clinical care of the COVID patients (n = 74, 36.5%), followed by 63 (31%) participants who would want to get involved in health education and awareness-raising activities. Out of 58 respondents unwilling to volunteer, lack of training and adequate skills was the most frequently reported reason for not willing to volunteer (n = 23, 40%) (Table 6).

**Table 6:** Preferred area of work and motivation to volunteer in willing participants and reasons for hesitancy in unwilling participants.

Preferred area of work and reasons for motivation and hesitancy	N	%
Preferred area of work in willing participants		
1. Clinical care of the patient	74	36.45
2. Health education/Awareness raising	63	31.03
3. Counseling of patients	22	10.84
4. Triage of patients	12	5.91
5. Online work of any type	12	5.91
6. Administrative duty	9	4.43
7. Contact tracing	6	2.96
8. Swab collection	5	2.46
Total	203	100
**Motivation behind volunteering**		
1. Opportunity to serve mankind	97	47.78
2. I will learn new things	69	34.00
3. This will add up to my CV/Resume (for future career opportunities including masters level education)	20	9.85
4. I can make new contacts and more friends	7	3.45
5. I expect a written recognition from government/concerned authorities	7	3.45
6. I want to be the next COVID hero	3	1.48
Total	203	100
**Reasons for hesitancy in unwilling participants:**		
1. Lack of training and adequate skills	23	39.66
2. Fear of transmission of infection to friends and family	19	32.76
3. My parents will not allow me	8	13.79
4. Lack of PPE and other safety equipment	6	10.34
5. Fear of own health	2	3.45
Total	58	100

## 4 Discussion

The majority of respondents in our study were willing to volunteer during the COVID-19 pandemic. According to a comparable survey, 98.7% of respondents said they were willing to obtain a medical history and do a physical examination [11]. The number of people who were willing to offer spontaneous support and help in COVID-19 accounted for 85.6% of the respondents in a Chinese study [12]. The prevalence of willingness in our study is considerably higher than previous studies conducted in Indonesia, where 2374 out of 4870 (48.7%) participants expressed their will to volunteer [13]. Similarly, we also observed a comparable lack of willingness in publications by Drexler et al. and Al Saif et al. [14,15]. However, a prior study among the Nepalese frontline health workers found that 64% were willing to work under challenging conditions [16].

Our research found that the willingness to participate was highest in final year students than in other years. However, a Chinese study showed the willingness to volunteer decreased with seniority [12]. Similarly, a report in Darussalam found that first-year students (72.4%) were more willing than second-year students (53.3%) (p < 0.001) and others [17]. Our study had a greater number of females due to the addition of nursing students. Findings with higher female respondents were found in studies from Brunei (77.8%) and in study by Nuwan et al. (n = 601, 70.2%) [17,18]. However, the findings from Brunei revealed that gender had no impact on nursing students' willingness to volunteer during a pandemic since males were just as likely as women to do so (p = 0.077) [17]. This is in contrast to our study and another study on Chinese medical students done by Yu et al. (2020), where females had comparatively higher willingness to volunteer than males [12].

The motivating elements in our study were found to be similar to others, such as a cross-sectional study from Hamburg, Germany, in which reasons for volunteering included a sense of obligation, social commitment, interest in medical activities, and skill growth [14]. In a Nigerian survey, two-thirds of respondents (66.4%) said they had a moral obligation to volunteer, and more than half of them (56.2%) said they would volunteer if the government asked them to Ref. [19]. Even in a Sri Lankan research, 804 out of 856 (93.9%) medical students reported that they have a duty to aid in the nation's COVID-19 response [18]. A study from Brazil also observed that a sense of duty and willingness to take risks were the vital factor that influenced the longing to volunteer during the pandemic [20].

In our survey, the most common reasons for people's hesitation to volunteer were a lack of appropriate skills and a fear of infecting their friends and relatives. Our findings are consistent with a study done in Indonesia, in which most students expressed concerns for potential health risks to themselves and their families as well as with the study conducted in Nigeria in which the participants would be willing to volunteer if provided with appropriate PPE and training [13,19].

The majority of the participants in our study chose clinical patient care, awareness activities, patient counseling, and triage as their top priority duties. Similar findings were also found by W. Cheah et al., D. Bazan et al. and Adejimi et al. [11,19,21].

Our study's strength comes from the inclusion of both MBBS and nursing students, as well as the use of proportionate stratified random sampling. As the study includes students from a single medical school and half of the participants from a military household, similar studies in other medical schools need to be conducted to generalize the findings.

## 5 Conclusion

Our study shows that, majority of participants are eager to volunteer and are particularly interested in giving clinical care to COVID-19 patients. However, adequate training and personal protective equipment are a must for effective volunteering by them in public health emergencies. We recommend comparable research at various medical colleges because of its relevance to national authorities and the healthcare system as a whole.

## Ethical approval

The study was approved by the Institutional Review Committee (IRC) of the Nepalese Army Institute of Health Sciences (NAIHS) with reference number 432.

## Sources of funding

None.

## Author contribution

Manoj Khadka (MK) and Swojay Maharjan (SM) were involved in the conceptualization of the study. Subodh Dhakal (SD), Lee Budhathoki (LB), and Parag Karki (PK) were involved in the data analysis. All authors (PK, LB, MK, SM, SD, SP, AP, PR, UR, SR) were involved in the design of the study, data collection, literature review, writing and editing the manuscript, and approved the final version of the manuscript.

## Registration of research studies

1. Name of the registry: Research Registry (http://www.researchregistry.com).

2. Unique Identifying number or registration ID: researchregistry7338.

3. Hyperlink to your specific registration (must be publicly accessible and will be checked): Research Registry.

## Guarantor

Mr. Swojay Maharjan, Nepalese Army Institute of Health Sciences, 44600 Kathmandu, Nepal. Email: maharjan.swojay@gmail.com, Phone: +977-9861284969.

## Consent

All the participants were informed about the study and its objectives during the time of data collection. Consent form was incorporated in the questionnaire itself.

## Funding

This study did not receive any grant from any funding agencies in public or organizations.

## Provenance and peer review

Not commissioned, externally peer-reviewed.

## Declaration of competing interest

No conflicts of interest.
